# Alzheimer’s disease and COVID-19: Interactions, intrinsic linkages, and the role of immunoinflammatory responses in this process

**DOI:** 10.3389/fimmu.2023.1120495

**Published:** 2023-02-09

**Authors:** Wei Li, Lin Sun, Ling Yue, Shifu Xiao

**Affiliations:** ^1^ Department of Geriatric Psychiatry, Shanghai Mental Health Center, Shanghai Jiao Tong University School of Medicine, Shanghai, China; ^2^ Alzheimer’s Disease and Related Disorders Center, Shanghai Jiao Tong University, Shanghai, China

**Keywords:** Alzheimer’s disease (AD), COVID-19, epidemiological studies, mechanism, inflammation and immune responses

## Abstract

Alzheimer’s disease (AD) and COVID-19 share many common risk factors, such as advanced age, complications, *APOE* genotype, etc. Epidemiological studies have also confirmed the internal relationship between the two diseases. For example, studies have found that AD patients are more likely to suffer from COVID-19, and after infection with COVID-19, AD also has a much higher risk of death than other chronic diseases, and what’s more interesting is that the risk of developing AD in the future is significantly higher after infection with COVID-19. Therefore, this review gives a detailed introduction to the internal relationship between Alzheimer’s disease and COVID-19 from the perspectives of epidemiology, susceptibility and mortality. At the same time, we focused on the important role of inflammation and immune responses in promoting the onset and death of AD from COVID-19.

## Introduction

Alzheimer’s disease (AD) is the most common type of dementia and one of the most common neurodegenerative diseases in the elderly, accounting for approximately 90% of dementia cases in this population. AD is characterized by irreversible and progressive loss of function, cognition, and behavior and is often accompanied by various brain disorders such as aphasia, agnosia, amnesia, and, apraxia (1). Its diagnosis is mainly based on clinical presentation as well as fluid and imaging biomarkers that meet several criteria (2), such as “AT(N)” (amyloid, tau, and neurodegeneration) (3, 4). AD is considered as a multifactorial disease, and two main hypotheses have been proposed for the cause of AD: the amyloid hypothesis and the cholinergic hypothesis (5). However, only a limited number of drugs have been developed to address this theory, and only two drugs have been approved for the treatment of AD, including N-methyld-aspartic acid (NMDA) antagonists and cholinesterase inhibitors, which are only effective against the symptoms of AD and do not cure or prevent the disease (5). According to the report of Alzheimer’s Disease International in 2018, the global prevalence of dementia was about 50 million people and was expected to triple by 2050, with two-thirds of them living in low - and middle-income countries (6). Although studies have shown that dementia rates are declining in high-income countries, the evidence is less convincing (7). Since the irreversibility of Alzheimer’s disease, it is extremely important to find out its influencing factors and make early intervention and treatment for its prognosis. The most serious risk factors for Alzheimer’s disease are advanced age and having at least one apolipoprotein E (APOE) epsilon 4 allele (8). Moreover, the Lancet also identifies twelve controllable risk factors such as less education, smoking, excessive alcohol consumption, physical inactivity, low social contact, obesity, depression, diabetes, hypertension, air pollution, hearing impairment and traumatic brain injury, which can affect the progression of dementia in 40 percent of patients (9). By contrast, healthy lifestyle choices such as physical exercise, leisure activities, and Mediterranean diet are considered protective against AD (10). In addition to these factors, the relationship between the novel coronavirus disease (COVID-19) and Alzheimer’s disease is receiving increasing attention. This review will explore the link between COVID-19 and Alzheimer’s disease in detail, including epidemiological investigations, interactions, and possible mechanisms.

## The novel coronavirus disease (COVID-19)

COVID-19 is a new infectious disease caused by the severe acute respiratory syndrome coronavirus type 2 (SARS-CoV-2). Since its discovery in December 2019 in mainland China, COVID-19 has swept the world, causing untold losses and casualties (11). On 11 March 2020, the World Health Organization (WHO) declared COVID-19 a “public health Emergency of International concern” (12). As of 27 June 2022, the COVID-19 pandemic has infected more than 540 million people worldwide and caused more than 6 million deaths (13). SARS-CoV-2 is an RNA virus whose genome contains single-stranded positive RNA within a membrane envelope with an average diameter of 75-150 nm (14). It belongs taxonomically to the coronavirus family and the sarbecvirus subgenus, which contains several other species that cause mild to severe human illness (15). SARS-CoV-2 is highly contagious (16), with an estimated reproductive number, R naught (R0), of between 1.4 and 5.6 (17). Once infected with the virus, it can activate innate and adaptive immune responses and lead to large-scale inflammatory responses in the later stages of the disease (18). SARS-CoV-2 is usually spread by respiratory droplets. The average incubation period is 6.4 days. Symptoms after infection usually include cough, fever, myalgia, fatigue and difficulty breathing, and the typical findings of chest computed tomography (CT) images for individuals with COVID-19 are multifocal bilateral patchy ground-glass opacities or consolidation with interlobular septal and vascular thickening in the peripheral areas of the lungs (19). While most patients experience mild illness, a small number develop severe hypoxia requiring hospitalization or mechanical ventilation, and the most severe outcomes even include death (20, 21). Although many treatment options are being explored (e.g., convalescent plasma, and traditional Chinese medicine (TCM)), no large-scale treatment is available. What’s worse, the development of a vaccine for COVID-19 has not been smooth and there have also been some reports of adverse reactions after receiving the vaccine (22). Currently, COVID-19 prevention relies largely on public health policies, such as physical distancing, travel restrictions and city lockdowns, which are also extremely ineffective. Therefore, the future prevention strategies must focus on the target population. The epidemiological evidence suggests that advanced age and complications are the greatest risk factors for poor prognosis in COVID-19 patients (12, 23), and they are also major risk factors for Alzheimer’s disease. There are various indications that COVID-19 and Alzheimer’s disease might be intrinsically related.

## COVID-19 increases the risk of developing Alzheimer’s disease in the future: Epidemiological evidence

Yu AT and Absar NM reported two patients diagnosed with COVID-19 who experienced prolonged infection after COVID-19 pneumonia and developed rapidly progressive dementia after a follow-up period of 5-10 months (22). An observational study in France reported that one-third of COVID-19 patients admitted to hospital with acute-respiratory-distress-Syndrome (ARDS) had evidence of cognitive impairment at discharge (24). Mcloughlin BC et al found that hospitalized COVID-19 patients who developed delirium during hospitalization had lower cognitive scores 1 month after discharge (25). Apple AC et al found that cognitive post-acute sequelae of SARS-CoV-2 (PASC) could occur after mild COVID-19 infection (26). Garrigues E et al found that four months after COVID-19 hospitalization, 30‐40% of patients reported problems with memory, attention, and sleep, while 10‐15% reported loss of taste and/or smell (27). Gordon MN et al pointed out that young people were also at risk of developing cognitive symptoms associated with COVID-19, even without severe illness (28). Frontera JA et al. have found that after infection with COVID-19, neurodegenerative biomarkers, such as beta-amyloid protein (aβ 40,42), total tau protein (t-tau), phosphorylated taU-181 (p-tau181), glial fibrillary acid protein (GFAP), neurofilament light chain (NfL), and ubiquitin carboxy-terminal hydrolase L1 (UCHL1), increased to levels observed in AD dementia, and was associated with encephalopathy and worse outcomes in hospitalized COVID-19 patients (29). Wang et al. reviewed 6,245,282 older adults (age ≥65 years), who visited the hospital between February 2020 and May 2021 and found that there was a significant increase in the risk of a new diagnosis of Alzheimer’s within 360 days of the initial diagnosis of COVID-19 (hazard ratio or HR:1.69, 95% CI: 1.53-1.72), especially in patients and women aged 85 years or older (30). A Mendelian randomization study indicated that hospitalization of COVID-19 was significantly associated with a higher risk of Alzheimer’s disease (OR: 1.02, 95% CI: 1.01-1.03, P: 1.19E-03); meanwhile, there was also a significant and positive genetic correlation between hospitalization of COVID-19 and AD (genetic correlation: 0.23, P = 8.36E-07) (31).

## Why does COVID-19 increase the risk of Alzheimer’s disease? (possible mechanisms)

### Genetic susceptibility

Apolipoprotein E is a major carrier of cholesterol in the central nervous system and an important component of very low-density lipoprotein (VLDL). Of its three alleles (epsilon 2, epsilon 3, and epsilon 4), individuals carrying the epsilon 4 allele are at a higher risk of AD, as *APOE E4* genotype will increase fibrinogenesis in the brains of Alzheimer’s patients (32). Genetic correlation analysis showed that there was a significant positive correlation between AD and the heredity of hospitalized COVID-19 (rg = 0.271) (33). Meanwhile, individuals with the *APOE E4 gene* have also been shown to have more severe cognitive impairment after infecting with AD COVID-19 (34).

### Nerve damage

More and more reports have shown that SARS-CoV-2 can alter the dense blood-brain barrier (BBB), enter the brain and damage neurons directly or indirectly, leading to long-term neurological sequelae, such as Alzheimer’s disease (35, 36). Some imaging studies have shown that after infection with COVID-19, patients may have low metabolism in the frontal lobe and cerebellum region, which will eventually lead to the apoptosis of nerve cell (37, 38). Moreover, COVID-19 is also known to infect the hippocampus and further infect the spinal cord, which can lead to further neurodegeneration due to misdirected host immune responses and/or direct damage to nerve cells through replication of viral particles, as in acute encephalitis (39).

### Angiotensin-converting enzyme 2 (ACE-2)

The action of ACE-2 is associated with the renin-angiotensin-aldosterone system (RAAS), which is the entry receptor of COVID-19 (40). Once the COVID-19 virus binds to the ACE-2 receptor, it leads to an increased production of the pro-inflammatory phase, which can lead to serious complications known as “cytokine storms.” (41) In the blood-brain barrier and the meninges that cover the spinal cord, the combination of the virus with ACE-2 weakens the enzyme’s ability to protect nerve tissue, thus making them more vulnerable to damage, leading to encephalitis, or myelitis (42). In addition, there has also been reported that the combination of the virus with ACE-2 in the cerebrovascular increased the intracavitary pressure, which eventually led to the patient’s cerebral hemorrhage (43).

## Why are people with dementia more susceptible to COVID-19? (epidemiological evidence)

As of September 21, 2021, a series of observational retrospective analyses using electronic health records (EHRs) from Columbia University Irvine Medical Center/New York-Presbyterian Hospital (CUIMC/NYP) have shown that Alzheimer’s disease was a major risk factor for COVID-19 infection (44). A matched-cohort study using primary care electronic records in the UK showed that patients with Down syndrome (DS) were more likely to be diagnosed with COVID-19 than controls (7.4% vs 5.6%, p≤0.001, odds ratio (OR) = 1.35; 95% CI = 1.23-1.48) (45).

A retrospective cohort study of 262,847 vaccinated older adults (age 73.8±6.81 years) in the United States between December 2020 and August 2021 showed that people with dementia had an increased risk of developing breakthrough infections compared to unvaccinated patients, with the highest odds for patients with Lewy body dementia (LBD) (adjusted odds ratio or AOR: 3.06, 95% confidence interval or CI [1.45 to 6.66]) (30). A survey of the Korean National Health Insurance Database suggested that COVID-19 patients were more likely to have a prediagnosed AD (adjusted odds ratio [aOR] = 2.11, 95% confidence interval [CI] = 1.79-2.50, p values < 0.001) compared to the control group (46). An observational cohort study showed that Alzheimer’s disease (OR = 2.29, 95% CI: 1.25-4.16) and dementia (OR = 2.16, 95% CI: 1.36-3.42) were the most significant risk factors for COVID-19 (47). A population-based cohort study conducted in the Lazio region has shown that Alzheimer’s disease was more susceptible to COVID-19, and being males and aged ≥85 years of age were at a higher risk of death from infection (48). A cross-sectional analysis showed that the preexisting cognitive impairment was significantly associated with a higher likelihood of COVID-19 infection (OR, CI: 1.51, 1.35-1.70) (49). A population-based risk assessment model for severe disease outcome from COVID-19 confirmed that Alzheimer’s disease was associated with the highest risk for hospitalization (aHR 3.19, CI: 2.88-3.52) and death (aHR 4.04, CI: 3.32-4.91) (50). A meta-analysis suggested that patients with dementia, including Alzheimer’s disease, had an increased risk of hospitalization and death after contracting COVID-19 (51). Moreover, several reviews have also indicated that neurodegenerative diseases, including Alzheimer’s disease, Parkinson’s disease and epilepsy, are major risk factors for COVID-19 infection, and demand extra care as well as improvised treatment (52).

## Why are people with dementia more susceptible to COVID-19? (possible mechanism)

There are several reasons why people with Alzheimer’s disease may be more susceptible to COVID-19. First, people with Alzheimer’s are often older, which is also a major risk factor for COVID-19 (53). It has been speculated that aging may induce the production of reactive oxygen species (ROS), increase neuroinflammation intensify, overproduction of Aβ, which contributes to the pathogenesis of COVID-19 and AD. Since aging is characterized by a progressive loss of blood-brain barrier integrity, older adults may be more susceptible to neuroaggression during the SARS-CoV-2 infection (54); Second, people with dementia often do not have access to accurate information and facts about the COVID-19 pandemic, and they may have difficulty remembering protective procedures or understanding public health messages sent to them. As a result, these people tend to be more susceptible to COVID-19 infection (55). Third, the reduced ability to cope with sudden changes in the social environment makes people with dementia more vulnerable to COVID-19 infection as well as poorer clinical and social outcomes (56). Fourth, people with dementia tend to have a variety of physical conditions, such as hypertension, diabetes, heart diseases, so the consequences of contracting COVID-19 are often worse (57). Fifth, elderly care institutions or medical institutions in many areas, such as China, often receive a large number of dementia patients with a very large population density. Once the COVID-19 infection occurs, the epidemic will spread rapidly. Sixth, some studies have suggested that Alzheimer’s disease and COVID-19 may share a common genetic structure (58), for example, four hub genes (ITPR1, ITPR3, ITPKB, RAPGEF3) were considered as important factors in the development of AD that were affected by COVID-19 (59). Homozygous *APOE e4e4* is not only a risk factor for AD, but also increases the susceptibility to severe infection of SARS-CoV-2 (60). Compared to APOE E3 mice, APOE2 and APOE4 mice exhibited increased viral loads as well as suppressed adaptive immune responses early after infection (61). Seventh, serum cholesterol binds to APOE receptors and induces ACE-2 receptor transport to the cell surface. Ultra-resolution imaging studies have shown that high cholesterol levels will confer a 2-fold in increase the entry site of SARS-CoV-2, thus facilitating the entry of the virus (62). At the same time, elevated Aβ levels, specifically Aβ1-42, are associated with SARS-CoV-2, and the binding of Aβ1-42 to the spike protein S1 subunit (S1) of SARS-CoV-2 and ACE2 may negatively affect the course and severity of SARS-CoV-2 infection (63).

## Why individuals with Alzheimer’s disease are most likely to die after contracting COVID-19?(epidemiological evidence)

According to relevant reports, people with dementia are more than three times more likely to die from COVID-19 than those without dementia and about 30% of COVID-19 deaths are in people with dementia (64, 65). A cohort study conducted in Tehran, Iran showed that Alzheimer disease were associated with a higher risk of death related to COVID-19 (66). A cohort study conducted in England showed that COVID-19 survivors with pre-existing dementia had a higher risk of hospitalization or death (age and gender adjusted HR 2.47, 1.37 to 4.44, p = 0.002) (67). According to a study by the UK Biobank, older people with Alzheimer’s disease were at the highest risk and mortality from COVID-19 (68). Another UK Biobank study involving 12,863 patients found that all-cause dementia and AD were age-independent risk factors for the severity and death of COVID-19 disease (69). A population-based register study has shown that there was a significant increase in mortality from diabetes and Alzheimer’s disease after infection with COVID-19 (70). In the study by Fathi M et al., they concluded that inpatients with Alzheimer’s disease had an increased risk for 28-day mortality from COVID-19 (71). In the study of Chung SJ et al., they found that AD was not associated with increased susceptibility to COVID-19 infection, but was associated with severe COVID-19 complications, especially mortality (72). Zhao Y et al noted that about 35% of COVID-19 patients present with neurological and neuropsychiatric symptoms, a previous diagnosis of Alzheimer’s disease (AD) predicts the highest risk of COVID-19, and elderly AD patients have the highest mortality rate (73). Wang et al. found a significant association between Alzheimer’s disease (AD) and increased risk of COVID-19 infection and mortality (30). Harb AA et al. found that hospitalized patients with COVID-19 dementia had a higher mortality rate, but dementia was not an independent risk factor for death (74). Moreover, a 93-country study also showed that there was a strong link between AD and COVID-19 deaths (75). Therefore, we are relatively certain that Alzheimer’s disease can increase the risk of death in patients with COVID-19.

## Why individuals with Alzheimer’s disease are most likely to die after contracting COVID-19? (immune and inflammatory mechanisms)

In addition to the effects of old age, comorbidities, and genetics described above, immune and inflammatory responses appear to play an important role in promoting AD death. However, there is little evidence to support SARS-CoV-2 infection of central nervous system cells, and most neurological symptoms appear to be due to hypoxia/ischemia and/or damage mediated by inflammatory damage (76). For example, Mao L et al found that COVID-19 can induce uncontrolled cytokine storms (mainly involving IL-6, IL-1β, and TNF), leading to a variety of symptoms including delirium (55). Ziff OJ et al found that there was a significant increased CSF proinflammatory cytokines, such as TNFɑ, IL-6, IL-1β, IL-8, among COVID-19 neurological patients and these proinflammatory cytokines were negatively correlated with sAPPɑ and sAPPβ (31). Poloni TE et al found that the brain infected with COVID-19 would show increased innate immunity and microglia enhancement (77). Ganji R et al. pointed out that COVID-19 could hijack the mitochondria of immune cells, replicate within the mitochondrial structure, disrupt mitochondrial dynamics and lead to cell death (78). Daugherty AM et al suggested that the interaction of inflammation and oxidative stress may initiate a self-propagating cascade that drives subsequent age-related decline (79). Chiricosta L et al. concluded that SARS-CoV-2 worsens AD by increasing neurotoxicity due to elevated levels of inflammation, beta-amyloid, and oxidative stress (80). D’Arrigo JS pointed that immune responses and excessive inflammation may accelerate the progression of inflammatory neurodegeneration in the brain, thereby increasing the likelihood of post-infection memory impairment and accelerating the progression of Alzheimer’s disease (81). Butler MJ found that even in these patients who have recovered from COVID-19 infection, peripheral inflammation may contribute to the progression of neurodegenerative diseases through neuroinflammatory mechanisms (82). Guasp M et al. demonstrated that SARS-CoV-2 infection may promote inflammatory processes by disrupting the blood-brain barrier (83). Reiken S et al. provided evidence linking SARS-CoV-2 infection with TGF-β signaling activation and oxidation overload (84). Naughton SX et al. pointed out that the immune response and excessive inflammation in COVID-19 might accelerate the progression of inflammatory neurodegeneration in the brain, and the elderly were more prone to severe consequences after infection with SARS-CoV-2 (85). An autopsy report also showed that the most typical pathological features of COVID-19 were a large number of t lymphocytes and microthrombus formation in the lung, and a high activation of brain stem-related microglia (86). In conclusion, central nervous system autoimmune cascades triggered by COVID-19 may occur through a variety of pathways, including molecular mimicry, epitope diffusion, bystander activation, autoantibody production, and effector B cell immobilization (87), and ultimately contribute to the death of AD patients. The past studies on Alzheimer’s disease and COVID-19 infection and mortality rate or disease progression and other major findings will be presented in **Table 1**.

**Table 1:** The past studies on Alzheimer’s disease and COVID-19 infection and mortality rate or disease progression and other major findings.

Epidemiological investigation			
Author	The title of the paper	Published journal	Date of publication
Matias-Guiu JA,et al	Death Rate Due to COVID-19 in Alzheimer’s Disease and Frontotemporal Dementia.	J Alzheimers Dis.	2020
Daugherty AM, et al	COVID-19 as a risk factor for Alzheimer’s disease and related dementia: A perspective from Detroit, MI	Psychiatry Res.	2020
Li J, et al	Resilience of Alzheimer’s Disease to COVID-19	J Alzheimers Dis.	2020
Wang Q, et al	COVID-19 and dementia: Analyses of risk, disparity, and outcomes from electronic health records in the US.	Alzheimers Dement	2021
Zhang Q, et al	COVID-19 Case Fatality and Alzheimer’s Disease.	J Alzheimers Dis.	2021
Zhou J, et al	Cognitive disorders associated with hospitalization of COVID-19: Results from an observational cohort study.	Brain Behav Immun	2021
Harrison SL, et al	Associations between COVID-19 and 30-day thromboembolic events and mortality in people with dementia receiving antipsychotic medications	Pharmacol Res.	2021
Manzo C, et al	Could COVID-19 anosmia and olfactory dysfunction trigger an increased risk of future dementia in patients with ApoE4?	Med Hypotheses	2021
Burns A, et al	COVID-19 and dementia: experience from six European countries.	Int J Geriatr Psychiatry	2021
Wang Y, et al	Preexisting Mental Disorders Increase the Risk of COVID-19 Infection and Associated Mortality.	Front Public Health	2021
Tahira AC, et al	Dementia is an age-independent risk factor for severity and death in COVID-19 inpatients.	Alzheimers Dement	2021
Pan AP,et al	SARS-CoV-2 Susceptibility and COVID-19 Mortality Among Older Adults With Cognitive Impairment: Cross-Sectional Analysis From Hospital Records in a Diverse US Metropolitan Area.	Front Neurol	2021
Wang L, et al	Association of COVID-19 with New-Onset Alzheimer’s Disease	J Alzheimers Dis	2022
Zhang H, et al	COVID-19 and the risk of Alzheimer’s disease, amyotrophic lateral sclerosis, and multiple sclerosis	Ann Clin Transl Neurol.	2022
Li C, et al	COVID-19 and risk of neurodegenerative disorders: A Mendelian randomization study.	Transl Psychiatry	2022
Chung SJ, et al	Association of Alzheimer’s Disease with COVID-19 Susceptibility and Severe Complications: A Nationwide Cohort Study	J Alzheimers Dis.	2022
Apple AC, et al	Risk factors and abnormal cerebrospinal fluid associate with cognitive symptoms after mild COVID-19.	Ann Clin Transl Neurol	2022
Wang Y,et al	Clinical outcomes of COVID-19 infection among patients with Alzheimer’s disease or mild cognitive impairment.	Alzheimers Dement	2022
Li S, et al	Excess deaths from Alzheimer’s disease and Parkinson’s disease during the COVID-19 pandemic in the USA	Age Ageing	2022
Gilstrap L, et al	Trends in Mortality Rates Among Medicare Enrollees With Alzheimer Disease and Related Dementias Before and During the Early Phase of the COVID-19 Pandemic.	JAMA Neurol	2022
Zerbo O, et al	Population-based assessment of risks for severe COVID-19 disease outcomes.	Influenza Other Respir Viruses	2022
Baranova A, et al	Causal effect of COVID-19 on Alzheimer’s disease: A Mendelian randomization study	J Med Virol	2023
Mechanisms			
Author	The title of the paper	Published journal	Date of publication
Ding Q, et al	Angiotensin-converting enzyme 2 (ACE2) is upregulated in Alzheimer’s disease brain.	bioRxiv	2020
Xiong N, et al	Severe COVID-19 in Alzheimer’s disease: APOE4’s fault again?	Alzheimers Res Ther	2021
Magusali N, et al	A genetic link between risk for Alzheimer’s disease and severe COVID-19 outcomes *via* the OAS1 gene.	Brain	2021
Finelli C	Metabolic Syndrome, Alzheimer’s Disease, and Covid 19: A Possible Correlation.	Curr Alzheimer Res	2021
Poloni TE, et al	COVID-19-related neuropathology and microglial activation in elderly with and without dementia.	Brain Pathol	2021
Chiricosta L, et al	SARS-CoV-2 Exacerbates Beta-Amyloid Neurotoxicity, Inflammation and Oxidative Stress in Alzheimer’s Disease Patients	Int J Mol Sci.	2021
MacIntosh BJ, et al	Brain structure and function in people recovering from COVID-19 after hospital discharge or self-isolation: a longitudinal observational study protocol	CMAJ Open.	2021
Wang H, et al	Possible immunity, inflammation, and oxidative stress mechanisms of Alzheimer’s disease in COVID-19 patients.	Clin Neurol Neurosurg.	2021
Kas A, et al	The cerebral network of COVID-19-related encephalopathy: a longitudinal voxel-based 18F-FDG-PET study	Eur J Nucl Med Mol Imaging	2021
Zhou Y, et al	Network medicine links SARS-CoV-2/COVID-19 infection to brain microvascular injury and neuroinflammation in dementia-like cognitive impairment.	Alzheimers Res Ther	2021
Sindona C, et al	NOX2 Activation in COVID-19: Possible Implications for Neurodegenerative Diseases.	Medicina (Kaunas)	2021
Reiken S, et al	Alzheimer’s-like signaling in brains of COVID-19 patients	Alzheimers Dement	2022
Fu Y, et al	Single-nucleus RNA sequencing reveals the shared mechanisms inducing cognitive impairment between COVID-19 and Alzheimer’s disease.	Front Immunol.	2022
Koźmiński P, et al	New Imaging Modality of COVID-19 Pneumonia Developed on the Basis of Alzheimer’s Disease Research.	Int J Mol Sci.	2022
Zhang H, et al	APOE interacts with ACE2 inhibiting SARS-CoV-2 cellular entry and inflammation in COVID-19 patients.	Signal Transduct Target Ther	2022
Denaro CA, et al	COVID-19 and neurodegeneration: The mitochondrial connection.	Aging Cell	2022
Wang Y, et al	The Golgi apparatus: Site for convergence of COVID-19 brain fog and Alzheimer’s disease?	Mol Neurodegener	2022
Ziff OJ, et al	Amyloid processing in COVID-19-associated neurological syndromes	J Neurochem	2022
Qiu S, et al	A genome-wide cross-trait analysis highlights the shared genetic structure between COVID-19 and Alzheimer’s disease.	J Infect	2022
Ostendorf BN, et al	Common human genetic variants of APOE impact murine COVID-19 mortality.	Nature	2022
Nuovo GJ, et al	The amplification of CNS damage in Alzheimer’s disease due to SARS-CoV2 infection.	Ann Diagn Pathol	2022
Wang F, et al	Analysis and Identification Genetic Effect of SARS-CoV-2 Infections to Alzheimer’s Disease Patients by Integrated Bioinformatics	J Alzheimers Dis	2022
Green R, et al	SARS-CoV-2 infection increases the gene expression profile for Alzheimer’s disease risk.	Mol Ther Methods Clin Dev	2022
Onisiforou A, et al	Systems Bioinformatics Reveals Possible Relationship between COVID-19 and the Development of Neurological Diseases and Neuropsychiatric Disorders.	Viruses	2022

## Advances in drug treatments for Alzheimer’s disease and COVID-19

Treatments for Alzheimer’s disease include cholinesterase inhibitors (ChEIs) (Donepezil, Rivastin, and Galanthamine) and metaxine. In addition, antidepressants and antipsychotics are also used to control patients’ behavior and psychiatric symptoms (88). Previous studies have shown that disruption of intracellular Ca2+ homeostasis is not only an upstream pathologic pathway for AD, but also for SARS-CoV-2 virus infection and COVID-19 replication (89). Therefore, lithium could be repurposed to treat patients with AD, especially those with COVID-19 (90). Ginkgo biloba extract (GbE) and bobalide (BB) are important bioactive components of Ginkgo biloba extract (GbE), which have been reported to show neuroprotective effects in AD *via* multiple mechanisms such as anti-oxidative activities, anti-excitotoxicity, and anti-inflammatory. Interestingly, ginkgolides and BB also appear to exhibit antiviral properties against COVID-19 by inhibiting the major proteases of severe acute respiratory syndrome coronavirus type 2 (SARS-CoV-2) (44). Increasing data suggest that Zn2+ metabolism may be involved in neurodegeneration, and the mechanisms may involve modulation of synaptic plasticity through ProSAP/Shank scaffold, neurotransmitter metabolism, and gut microbiota. Moreover, Zn2+ has also been shown to be a potential adjuvant therapy in management of novel coronavirus infection (COVID-19) (91). Myricetin (MYR) is a flavonoid compound widely found in many natural plants, including the bayberry. MYR can enhance immune regulation function, inhibit cytokine storm, improve cardiac dysfunction, and has antiviral potential. So it can be used as an adjuvant treatment for Alzheimer’s disease, cardiovascular injury and other neurological diseases, and may be a potential drug against COVID-19 and other viral infections (92). Flavone luteolin is an important natural polyphenol present in several plants that show antioxidant, anticancer, cytoprotective, anti-inflammatory, and macrophage polarization effects. Recent reports suggest that flavone luteolin can inhibit systemic and neuroinflammatory responses in COVID-19 (93). Other promising treatments for AD and COVID-19 also include (biobased) lipid nanocarrier (81), inflammasome and JaK inhibitors (94), angiotensin converting enzyme (ACE) inhibitors (95), and Metformin (96). However, none of the above drugs have undergone large-scale clinical trials, and the future application prospects are unknown.

## Conclusions

There is a strong correlation between Alzheimer’s disease and COVID-19. The two factors influence each other, promote each other and ultimately lead to poor prognosis. In this process, inflammation and immune response are likely to play an extremely important role. In the future epidemic prevention process, we should focus on the protection of the above-mentioned population, so as to reduce the mortality of patients to the greatest extent.

## Author contributions

WL and LY contributed to the study concept and design. WL and LS wrote this wrote this article. SX provided the funding support. All authors contributed to the article and approved the submitted version.
